# A Medical Causerie

**Published:** 1915-11-13

**Authors:** 


### A MEDICAL CAUSERIE.

The value of an official endorsement is at a premium nowadays, and hence it is satisfactory to find that the Central War Committee has adopted a number of the suggestions which have been advanced in these columns. Addressing the governing bodies of the voluntary hospitals, the Committee points out that resident appointments ought not, in the present emergency, to be held by young men eligible for military service; that the honorary staff should take a larger measure of direct responsibility for the clinical work; and that where other help cannot be obtained, members of the staff should take "orderly" duty as resident officers. No doubt it is not pleasant for senior men to leave the domestic circle and to return for a time to emergency hospital calls, but here is their chance for taking a share in the "sacrifice" which is rightly claimed from juniors and seniors alike. Not less important is the suggestion that newly-qualified men may receive commissions in the R.A.M.C. and be at the disposal of the War Office on forty-eight hours' notice while holding resident appointments for a three months' term in a general hospital. This arrangement has been put into force at a certain limited number of the hospitals, but the reason for this limitation has never been apparent, and The Hospital has repeatedly urged that the plan should be enlarged. It is all to the good that this proposal is now being considered, as also the suggestion that senior students may act as dressers in hospitals other than those attached to medical schools, and may count the time so spent as valid for graduation purposes. This latter scheme was somewhat abruptly refused by some of the diploma-granting bodies when it was proposed by the West London Hospital; but here, as in other directions, the claims of the national situation are breaking down technical restrictions and monopolies.

The meetings of the General Medical Council hardly attract the attendance of many medical practitioners, and, therefore, there will not be much personal concern in the announcement that the new building in Hallam Street will offer greater accommodation than the well-known 299 Oxford Street. Still, there is some altruistic satisfaction in the knowledge that our pastors and masters are comfortably housed, even though we do not attend their debates. The excellent address of the President is an effective reminder of the wide range of interests which medicine in its public and official relations has to consider and of the burden of administrative responsibility which the Council has to sustain. A few years ago there was a good deal of fault-finding because the Council did not do something to put down unqualified practice, and was not actively concerned with the hard lot of the humble practitioner. But there is now a more general recognition that these undertakings are outside the duty and power of the Council, which exists much more in the public interest than in the interest of the profession. Its powers are constructed to this end, and to complain that it does not do a number of things which are quite outside its authority is only to show ignorance of its genesis and responsibility. That the profession has now a certain number of direct representatives on the Council does at first sight suggest that the opinions and wishes of the profession have to be considered, but the presence of these gentlemen does not alter the powers of the Council, and these are strictly defined by the Act of Parliament which called it into existence.

The intimation that Sir Thomas Fraser is to retire from the Council, after representing the University of Edinburgh for a period of ten years, will be received with regret not only by Edinburgh graduates, but by many others. In addition to his professional and scientific distinction, Sir Thomas has excellent organising ability and a fine gift of lucid and incisive speech. Obviously, he must have taken a prominent part in the revision of the Pharmacopœia, and his work in this direction has received a special compliment from the Chairman of the Committee. It is announced that the new edition has already reached a circulation of some 25,000 copies, though whether all the subscribers have purchased it gladly may be doubted. The fact is that the volume has a compulsory circulation, as every practising pharmacist must provide himself with a copy, not to speak of those who wish to bring their manuals of materia medica up to date. Therefore, whether good, bad, or indifferent, the Pharmacopœia is always sure of a large constituency, and in this respect its success needs no prophet.

Sir Clifford Allbutt has recently given an amusing account of an attempt on the part of himself and some of his colleagues to simplify the Pharmacopœia by the expulsion of substances which the agitators considered unnecessary. Without hoping for a reduction to the "half dozen" which, according to tradition, the late Sir Samuel Wilks declared to be all that was necessary, a preliminary attack was made upon what may be called duplicates. Thus, in face of the virtues of gentian and calumba as stomachic tonics, it was urged that chiretta might be dropped. But no sooner was the proposal made than one, or more, of the assembled physicians rose more in sorrow than in anger to declare that such an elimination would mean the removal of a remedy in which he, or they, had long trusted, and that, whatever else went by the board, chiretta at least must remain; other proposals on the same lines met a similar fate. Thus it has always been. Everyone agrees that it would be an advantage to cut down the number of the Pharmacopœial list, but when any particular substance is named there is always someone to stand up in defence of the threatened drug. And, after all, this does not matter very much, for the purpose of the Pharmacopœia is to secure that what physicians prescribe is interpreted by the pharmacist in the sense which they intend it.

